# Cortisol secretory patterns in deep and moderate neuromuscular blockades in laparoscopic surgery under total intravenous anesthesia: A prospective, single-blinded, randomized controlled trial

**DOI:** 10.1097/MD.0000000000030702

**Published:** 2022-09-30

**Authors:** Jeongyoon Lee, Jihyun An, Dong Hwan Lee, Jihyang Lee, Eunju Kim, Kyeongyoon Woo, Kyeong Hyo Kim

**Affiliations:** a Department of Anesthesiology and Pain Medicine, Daegu Fatima Hospital, DaeguKorea.

**Keywords:** adrenocorticotropic hormone, cortisol, enhanced recovery after surgery, laparoscopic surgery, neuromuscular blockade, rocuronium

## Abstract

Neuroendocrine stress response induces physiological changes depending on the type of surgery and anesthesia. Although the optimal depth of neuromuscular blockade for reducing this response remains unknown, deep neuromuscular blockade is known to improve the surgical environment. Therefore, we hypothesized that a patient’s stress response would be lower in surgical procedures under deep neuromuscular blockade than under moderate neuromuscular blockade.

**Methods:**

This prospective, randomized, single-blind study enrolled 72 patients who underwent laparoscopic gynecological surgery under general anesthesia and were assigned to group D (deep blockade: target train-of-four 0 and posttetanic count ≥ 1) or group M (moderate blockade: target train-of-four count 1–3). The primary endpoints were changing patterns in cortisol and adrenocorticotrophic hormone levels; the secondary endpoints were patient outcomes, such as hemodynamic variables, serum glucose level, postoperative pain in the postanesthesia care unit, and hospital stay.

**Results:**

The baseline characteristics were comparable between the 67 patients included in the 2 groups (34 in group M and 33 in group D). Cortisol and adrenocorticotrophic hormone levels increased perioperatively in both groups but without significant intergroup differences. Serum glucose level increased perioperatively and decreased postoperatively, but without a significant intergroup difference. Postoperative pain, fentanyl requirement in the postanesthesia care unit, and hospital stay were also comparable.

**Conclusions:**

Compared with moderate neuromuscular blockade, deep neuromuscular blockade improved the surgical environment without significant intergroup differences in the hormonal stress response.

## 1. Introduction

The stress response to surgery is a systemic reaction that can induce metabolic, immunological, and neurohormonal changes. A series of stress responses act on patients through various mechanisms by increasing the levels of adrenocorticotrophic hormone (ACTH), cortisol, and catecholamine as a neuroendocrine reaction; proteolysis and hyperglycemia as a metabolic reaction; and inhibition of the natural killer cell and T-helper cell functions as an immunologic reaction.^[1]^ These endocrine disturbances are affected by the severity of surgery,^[2]^ type of surgery,^[3]^ and type of anesthesia.^[4–6]^ Previous studies have investigated anesthesia methods that reduce stress response. Studies have shown that stress response is lower in total intravenous anesthesia (TIVA) than in sevoflurane anesthesia^[5]^ and in regional anesthesia than in general anesthesia.^[6]^ However, few studies exist on the appropriate depth of neuromuscular blockade in relation to the stress response. Compared with moderate neuromuscular blockade, deep neuromuscular blockade improves surgical conditions and offers advantages such as a better surgical visual field,^[7,8]^ reduced bleeding during surgery,^[9]^ and reduced postoperative pain.^[9,10]^ Therefore, we hypothesized that performing surgeries under deep neuromuscular blockade rather than moderate neuromuscular blockade could reduce the patient’s stress response. To this end, this study investigated whether the maintenance of anesthesia through deep neuromuscular blockade rather than moderate neuromuscular blockade by utilizing neuromuscular monitoring offered an advantage against the hormonal response of the hypothalamic–pituitary–adrenal axis in the American Society of Anesthesiologists (ASA) class 1 to 2 patients undergoing laparoscopic gynecological surgery. The primary endpoints were changes in cortisol and ACTH levels, and the secondary endpoints were patient outcomes such as hemodynamic variables, neuromuscular recovery time, postoperative pain, serum glucose level, surgeon satisfaction scores, and hospital stay.

## 2. Methods

### 2.1. Ethics

This prospective, randomized, single-blind study was approved by ethics committee of Daegu Fatima Hospital (approval number: IRB DFH19MRIO385). After obtaining written informed consent, 72 patients with ASA physical status 1 or 2, aged 20 to 70 years, and scheduled for laparoscopic gynecological surgery were included in this study. The procedures were conducted in line with the principles of the Declaration of Helsinki, 2013.

### 2.2. Study protocol

To reduce the number of variables pertaining to the surgical method, the surgery was performed by 1 surgeon who was blinded to the patients’ group allocation, and 1 anesthesiologist managed anesthesia from induction to emergence. The exclusion criteria were diseases that affected the sympathetic response and hormonal secretion, such as thyroid disease, renal disease (creatinine level >1.5 mg/dL), diabetes mellitus, history of steroid or associated drug use, alcohol abuse, neuropsychiatric disease, and body mass index (BMI) of >25 kg/m^2^. The patients were randomly assigned to group D (deep neuromuscular blockade) or group M (moderate neuromuscular blockade) using the closed envelope technique.

All surgeries were performed between 8:30 am and 10:30 am to reduce circadian variations in the levels of the circulating stress hormone cortisol. Anesthesia was performed using TIVA (target-controlled infusion of propofol, Marsh pharmacokinetic model, and remifentanil, Minto pharmacokinetic model) under blood pressure (using a 22-gauge radial artery catheter), heart rate, electrocardiogram, oxygen saturation, bispectral index, and neuromuscular monitoring (TwitchView Monitor, Blink Device Company, Seattle, WA).

For electromyography-based neuromuscular monitoring, stimulating electrodes were placed over the patient’s ulnar nerve and sensing electrodes were placed over the adductor pollicis muscle and first dorsal interosseus muscle. Before neuromuscular blockade induction, calibration was performed. Train-of-four (TOF) count and posttetanic count were measured every 5 minutes during anesthesia.

After the loss of consciousness, patients in each group were administered rocuronium intravenously (0.6 mg/kg [moderate neuromuscular blockade] to 1.2 mg/kg [deep neuromuscular blockade]). Endotracheal intubation was performed when the TOF count reduced to 0 in both groups.

During surgery, the TOF counts of 1 to 3 in patients with moderate neuromuscular blockade and TOF count of 0 and posttetanic count of ≥1 in patients with deep neuromuscular blockade were maintained by intravenous administration of bolus rocuronium (5–10 mg). Moreover, when additional administration was requested by the operator, the number of times was recorded.

The propofol dose was adjusted to maintain the bispectral index value at 40 to 60. Intraoperative mean arterial pressure was maintained within 60 to 110 mm Hg and 30% of the baseline. Hypotension or hypertension was defined as out of range. If hypotension or hypertension persisted for >5 minutes, phenylephrine (100 μg/mL) or nicardipine (1 mg/mL) was administered. At emergence, neuromuscular blockade in groups M and D were reversed with 2 and 4 mg/kg sugammadex, respectively. Extubation was performed when consciousness and spontaneous respiration were sufficient under TOF monitoring. Recovery time was recorded by measuring the time interval between sugammadex administration and extubation. After transfer to the postanesthesia care unit (PACU), fentanyl was used for pain control, and if the postanesthesia recovery score was 9 or higher, the patient was discharged. Arterial blood sampling was performed 3 times: before surgery (T0: baseline), at the end of surgery (T1), and 1.5 hours after surgery (T2). After surgery was completed, the surgeon, blinded to the patients’ group allocation, evaluated the condition of the surgical field using a numeric rating scale (NRS) ranging from 1 to 10 points (1 point: poor; 10 points: great). Cortisol, ACTH, and serum glucose levels were measured using laboratory tests. Hemodynamic variables, estimated blood loss, recovery time from neuromuscular blockade, postoperative pain (NRS score), hospital stay, and mortality were also recorded as postoperative outcomes.

### 2.3. Statistical analyses

The appropriate sample size was calculated by defining the level of statistical significance as 0.05, α as 0.05, and β as 0.2 using the F test of G*power (www.psycho.uni-duesseldorf.de/abteilungen/aap/gpower3, Germany, version 3.1.9.4) based on the cortisol levels measured in a pilot study. The mean cortisol levels in groups D and M were 20 and 25 µg/dL, respectively, and the standard deviation was 7. The sample size calculated based on an effect size of 0.357 was 32 persons in each group. After considering a possible dropout rate of 10%, we included 36 persons in each group.

Statistical analyses were performed using IBM SPSS Statistics for Windows, version 21.0 (IBM Corp., Armonk, NY). Student *t* test was performed for between-group comparisons of age, weight, height, anesthesia time, operation time, rocuronium dose, remifentanil dose, propofol dose, sugammadex dose, fentanyl dose in the PACU, hospital stay, and fluid input and output. The Mann–Whitney *U* test was performed to evaluate the NRS score of the surgical site and the surgeon satisfaction score of the surgical field. Repeated-measures analysis of variance with Bonferroni correction for multiple comparisons was used to assess heart rate, mean blood pressure, serum glucose level, serum cortisol level, serum ACTH level, and serum ACTH-to-cortisol ratio.

## 3. Results

**Figure 1.**
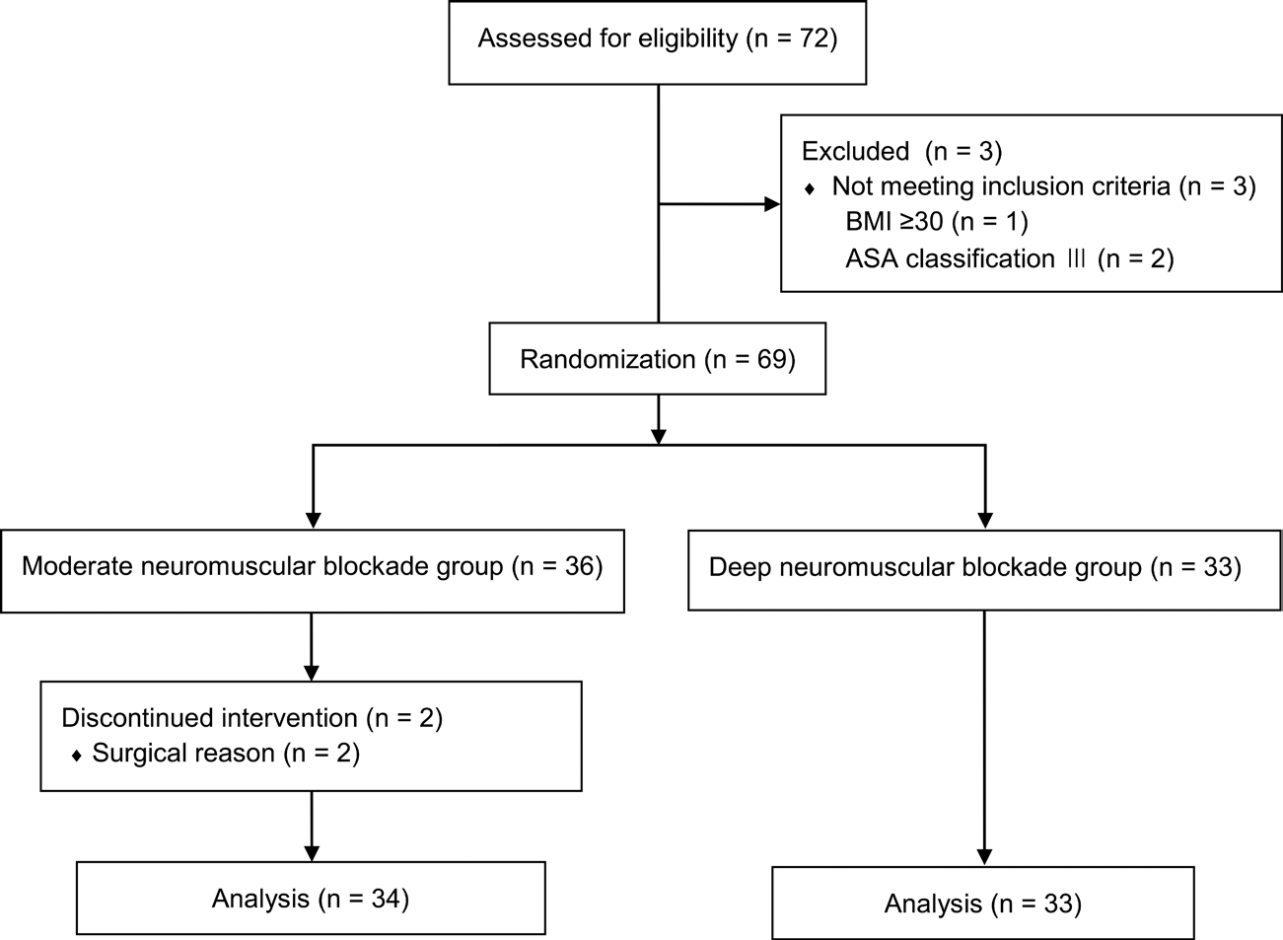
Flow diagram showing patient flow according to the study protocol. ASA = American Society of Anesthesiologists, BMI = body mass index.

**Figure 2.**
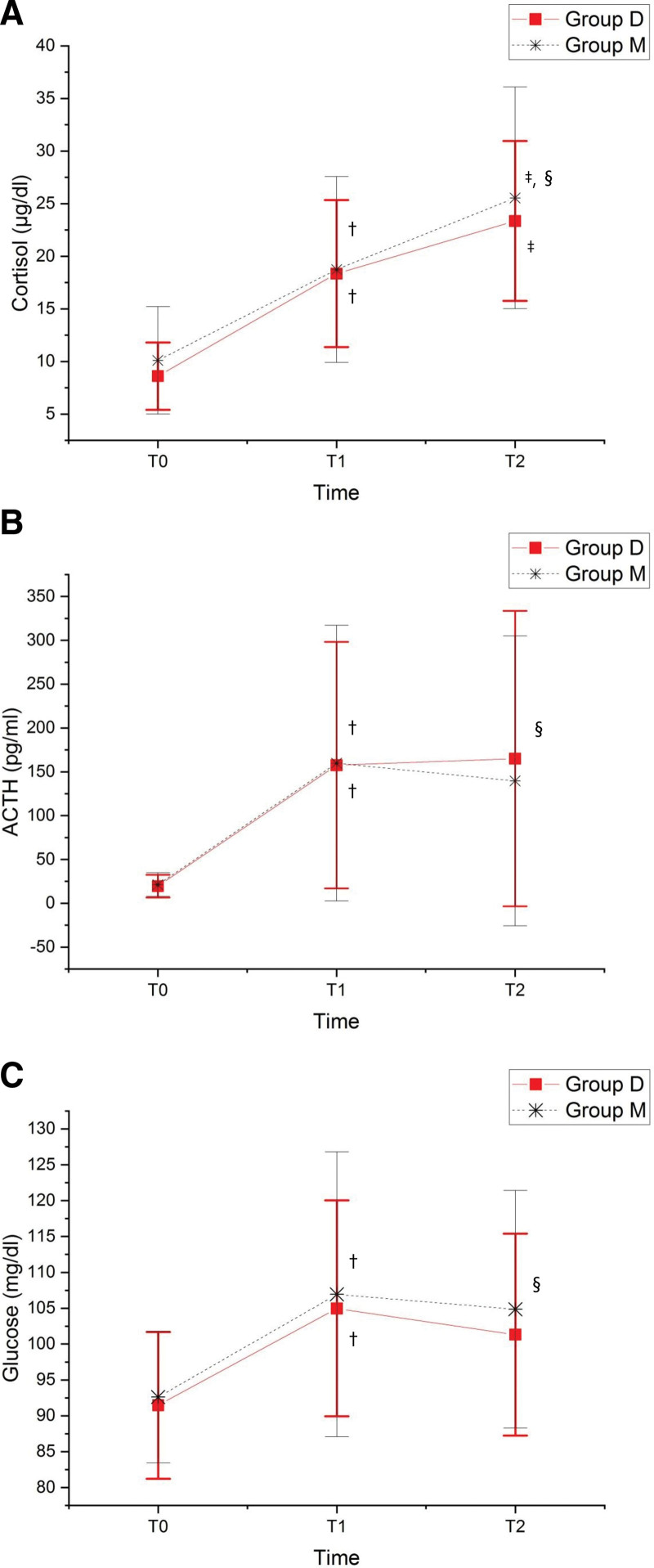
Comparison of stress hormone and glucose levels between the moderate and deep neuromuscular blockade groups. (A) Changes in serum cortisol levels. (B) Changes in serum ACTH levels. (C) Changes in serum glucose levels. ACTH = adrenocorticotropic hormone, Group D = deep neuromuscular blockade, Group M = moderate neuromuscular blockade, T0 = before surgery, T1 = end of surgery, T2 = 90 min after the surgery. ^†^*P* < .01 compared with T0 within group. ^‡^*P* < .01 compared with T1 within group. ^§^*P* > .05 between 2 groups.

The demographic and clinical characteristics of the patients are summarized in Tables 1 and 2. In total, 72 patients were included in the study and 2 patients in group M were excluded because their surgery was switched from laparoscopic surgery to open surgery. In addition, 2 patients were excluded because they were classified as having ASA class 3 and 1 patient because of a BMI of ≥30 kg/m^2^. Therefore, the study results were analyzed for 67 patients: 34 in group M and 33 in group D (Fig. 1). No significant differences were observed in the baseline characteristics such as age, height, weight, BMI, ASA score, and surgery type between the 2 groups. The doses of propofol and remifentanil administered to maintain anesthesia were also similar between the groups. Although a significant difference was observed in the dose of rocuronium used for each target depth of neuromuscular blockade, no difference was found in the recovery time taken for neuromuscular blockade reversal using sugammadex. Cortisol and ACTH levels were higher at T1 (end of surgery) and T2 (1.5 hours after surgery) than at T0 (before surgery) in both groups, but no significant difference was observed between the 2 groups (Fig. 2). Serum glucose level increased during surgery (T1) and decreased after surgery (T2), but no significant intergroup difference was observed (Fig. 2). After surgery, the surgical site pain NRS scores and fentanyl requirement in the PACU were comparable between the groups, and no intergroup difference was observed in hospital stay. However, additional administration of neuromuscular blocking agents at the surgeon’s request or sudden patient movement during surgery was significantly more frequent in group M than in group D (8/34 vs 0/33). Furthermore, after surgery, the surgeon’s satisfaction with the surgical field was better in group D than in group M (*P* < .001).

**Table 1:** Demographic and clinical characteristics of all the enrolled patients.

	Moderate blockade (n = 34)	Deep blockade (n = 33)	*P* value
Age (yr)	44.2 ± 9.7	40.7 ± 9.35	.14
Height (cm)	160.6 ± 4.8	160.6 ± 6.2	.97
Weight (kg)	59.5 ± 8.2	59.0 ± 11.8	.84
ASA physical status (1/2/3)	14/20/0	14/19/0	.92
Type of surgery			.52
Hysterectomy (n)	11	7	
Myomectomy (n)	13	14	
Cystectomy (n)	8	11	
Salpingo-oophorectomy (n)	2	1	
Anesthesia time (min)	115.09 ± 29.86	103.61 ± 27.71	.11
Operation time (min)	72.85 ± 25.82	60.74 ± 23.59	.05
Recovery time(min)	112.09 ± 68.16	134.97 ± 62.86	.16
Rocuronium dose (mg)	52.94 ± 21.84	76.26 ± 22.18	.000
Remifentanil dose (µg)	562.40 ± 320.90	528.20 ± 220.10	.61
Propofol dose (mg)	857.00 ± 252.33	786.76 ± 259.47	.27
Hypertension (n)	4	2	.43
Hypotension (n)	1	1	1.00
Additional rocuronium administration (n)	8	0	

Values are mean ± SD or numbers of patients (n).

ASA = American Society of Anesthesiologists, SD = standard deviation.

**Table 2:** Demographic and clinical characteristics of the patients in groups M and D.

	Group M (n = 34)	Group D (n = 33)	*P* value
Estimated blood loss (mL)	50.90 ± 61.37	37.06 ± 46.77	.30
Surgical pain (NRS)	5 (3.5, 7)	5 (3, 6)	.21
Fentanyl dose in PACU (µg)	90.30 ± 19.60	82.35 ± 27.20	.17
Satisfaction of surgeon	5 (4, 5)	5 (5, 6)	.001^*^
Hospital stay (d)	5 (5, 5)	5 (5, 5)	.81

Values are presented as mean ± SD and median (Q1, Q3).

Group D = deep neuromuscular blockade, Group M = moderate neuromuscular blockade, NRS = numerical rating scale scores (1–10; 1 = poor, 10 = good), PACU = postanesthesia care unit, SD = standard deviation.

* Surgeon’s satisfaction with the surgical field was higher in group D than in group M.

## 4. Discussion

The present study showed that deep neuromuscular blockade and moderate neuromuscular blockade had comparable effects on the stress response in patients undergoing laparoscopic gynecological surgery, but deep neuromuscular blockade had an advantage in improving the surgical environment. Stress response induces endocrine disturbances in patients undergoing surgery and is associated with patient outcomes. The Enhanced Recovery After Surgery program has also attempted to reduce stress response to improve patient outcomes.^[11,12]^ According to a previous study, stress response was lower with TIVA using propofol than with sevoflurane anesthesia, with significantly decreased ACTH, cortisol, and growth hormone levels.^[5]^ In addition, in laparoscopic cholecystectomy, spinal anesthesia resulted in lower serum cortisol levels than general anesthesia,^[6]^ and deep propofol anesthesia was advantageous over light anesthesia.^[13]^

Studies on deep neuromuscular blockade have reported improved surgical visual fields,^[7,8]^ reduced intraoperative bleeding,^[9]^ and reduced postoperative pain.^[9,10]^ Therefore, we expected that if deep neuromuscular block offers an advantage in the surgical procedure, it could reduce stress response and improve patient outcomes. ACTH and cortisol levels reflect the degree of stress during surgery,^[14,15]^ and cortisol levels are maximum after surgery.^[16,17]^ Cortisol and ACTH levels particularly increased during the reversal and recovery phases of anesthesia, but not during surgical manipulation, suggesting that stress response may be more closely related to anesthesia than to the surgery itself.^[16]^ In this study, ACTH and cortisol levels increased as the surgery progressed, but no significant difference was found between groups D and M. These results suggest that neuromuscular blockade is not a major factor for stress response. Therefore, maintenance of general anesthesia through moderate neuromuscular block is sufficient considering stress response.

Serum glucose level is also related to surgical infection and mortality after surgery, one of the main factors considered in the Enhanced Recovery After Surgery program, regardless of the presence or absence of diabetes.^[11,12]^ Moreover, serum glucose level is closely related to the cortisol level, and surgical stress can induce insulin resistance and hyperglycemia by promoting blood sugar production.^[1]^ Furthermore, a previous study revealed that deep propofol anesthesia had an advantage over light propofol anesthesia in glycemic control.^[13]^ Although both study groups showed a tendency toward an increase in serum glucose levels as the surgery progressed, no significant difference was found in glucose levels between the 2 groups. Nevertheless, because patients with diabetes were excluded from this study, further studies including patients with a history of diabetes, who are more affected by blood sugar control during surgery, are warranted to confirm these findings.

Previous studies have also reported that deep neuromuscular blockade reduces postoperative pain to a greater level than moderate neuromuscular blockade.^[9,10]^ However, in this study, we observed no significant difference between the 2 groups in terms of postoperative pain score and the dose of opioids administered for analgesia in the PACU, and no significant differences were observed in hospital stay.

Considering the surgeon’s satisfaction with the surgical field, group D had significantly higher scores than group M. The pressure of the abdominal cavity (pneumoperitoneum) was equally applied at 12 mm Hg in both groups, and it was expected that an appropriate surgical visual field would be secured; however, differences still existed in the surgeon’s satisfaction with this field. The average operation time was approximately 10 minutes shorter in group D than in group M. Therefore, deep neuromuscular blockade can be considered to improve the surgical environment. Nevertheless, these effects did not reduce the stress response. Moreover, Koo et al^[18]^ reported no significant difference in the levels of inflammatory mediators such as interleukin-6, tumor necrosis factor-α, and C-reactive protein in gastrectomy performed under deep neuromuscular blockade or moderate neuromuscular blockade.

This study has several limitations. The stress response is influenced by various factors. Although several variables were controlled for in this study, there may be other factors that were not thought to be affected by physiological and external factors. In addition, no standardized indicator to measure the stress response due to surgery is currently available, and in this study, it was investigated at the hormonal level that can induce physiological changes. Cortisol and ACTH levels were measured only at a specific time point owing to circadian variations in their levels, and a particular trend could be confirmed, but a possibility exists that the hormonal change was underestimated because the baseline (T0) levels measured in the morning were relatively high. Although no significant difference was observed in the classification of surgeries, patients in group D underwent a relatively simpler and less time-consuming ovarian cystectomy, which may have influenced the interpretation of the results. In addition, during the study, we closely monitored the neuromuscular blockade so that moderate neuromuscular blockade could be maintained more thoroughly than in actual clinical practice. In clinical settings, frequent additional administration of neuromuscular blocking agents may be required, and the patient’s stress response would likely be higher. Furthermore, we only checked the values at 3 disconnected time points, and no long-term follow-up was performed. Hence, the interpretation of the results is limited. If the results are analyzed through long-term follow-up at more continuous time intervals, more meaningful results regarding patient outcomes may be obtained.

In conclusion, although deep neuromuscular blockade can improve the surgical environment, its benefits on the stress response could not be confirmed. Therefore, moderate neuromuscular blockade performed under appropriate neuromuscular monitoring could reduce the costs associated with deep neuromuscular blockade. These findings highlight the need for further research on the depth of neuromuscular blockade in more large-scale and long-term studies.

## Acknowledgments

The authors would like to thank Editage (www.editage.co.kr) for English language editing.

## Author contributions

**Conceptualization:** Jeongyoon Lee, Jihyun An, Dong Hwan Lee

**Data curation:** Jeongyoon Lee, Dong Hwan Lee, Kyeong Hyo Kim

**Formal analysis:** Jeongyoon Lee, Jihyun An, Kyeong Hyo Kim

**Methodology:** Jihyun An, Dong Hwan Lee

**Supervision:** Jihyun An, Jihyang Lee, Eunju Kim, Kyeongyoon Woo

**Writing—Original draft:** Jeongyoon Lee

**Writing—review & editing:** Jeongyoon Lee, Jihyun An, Jihyang Lee, Eunju Kim, Kyeongyoon Woo, Kyeong Hyo Kim

## References

[1] Desborough JP. The stress response to trauma and surgery. Br J Anaesth. 2000;85:109–17. 10927999 10.1093/bja/85.1.109

[2] Marana R Margutti F Catalano GF Marana E. Stress responses to endoscopic surgery. Curr Opin Obstet Gynecol. 2000;12:303–7. 10954151 10.1097/00001703-200008000-00007

[3] Marana E Scambia G Maussier ML. Neuroendocrine stress response in patients undergoing benign ovarian cyst surgery by laparoscopy, minilaparotomy, and laparotomy. J Am Assoc Gynecol Laparosc. 2003;10:159–65. 12732764 10.1016/s1074-3804(05)60291-5

[4] Kelbel I Weiss M. Anaesthetics and immune function. Curr Opin Anaesthesiol. 2001;14:685–91. 17019166 10.1097/00001503-200112000-00015

[5] Marana E Colicci S Meo F Marana R Proietti R. Neuroendocrine stress response in gynecological laparoscopy: TIVA with propofol versus sevoflurane anesthesia. J Clin Anesth. 2010;22:250–5. 20522354 10.1016/j.jclinane.2009.07.011

[6] Das W Bhattacharya S Ghosh S Saha S Mallik S Pal S. Comparison between general anesthesia and spinal anesthesia in attenuation of stress response in laparoscopic cholecystectomy: a randomized prospective trial. Saudi J Anaesth. 2015;9:184–8. 25829908 10.4103/1658-354X.152881 PMC4374225

[7] Martini CH Boon M Bevers RF Aarts LP Dahan A. Evaluation of surgical conditions during laparoscopic surgery in patients with moderate vs deep neuromuscular block. Br J Anaesth. 2014;112:498–505. 24240315 10.1093/bja/aet377

[8] Staehr-Rye AK Rasmussen LS Rosenberg J. Surgical space conditions during low-pressure laparoscopic cholecystectomy with deep versus moderate neuromuscular blockade: a randomized clinical study. Anesth Analg. 2014;119:1084–92. 24977638 10.1213/ANE.0000000000000316

[9] Kang WS Oh CS Rhee KY. Deep neuromuscular blockade during spinal surgery reduces intra-operative blood loss: a randomised clinical trial. Eur J Anaesthesiol. 2020;37:187–95. 31860601 10.1097/EJA.0000000000001135

[10] Kim MH Lee KY Lee KY Min BS Yoo YC. Maintaining optimal surgical conditions with low insufflation pressures is possible with deep neuromuscular blockade during laparoscopic colorectal surgery: a prospective, randomized, double-blind, parallel-group clinical trial. Med (Baltimore). 2016;95:e2920. 10.1097/MD.0000000000002920 PMC4782877 26945393

[11] Carli F. Physiologic considerations of enhanced recovery after surgery (ERAS) programs: implications of the stress response. Can J Anaesth. 2015;62:110–9. 25501695 10.1007/s12630-014-0264-0

[12] Ren L Zhu D Wei Y. Enhanced recovery after surgery (ERAS) program attenuates stress and accelerates recovery in patients after radical resection for colorectal cancer: a prospective randomized controlled trial. World J Surg. 2012;36:407–14. 22102090 10.1007/s00268-011-1348-4

[13] Jung SM Cho CK. The effects of deep and light propofol anesthesia on stress response in patients undergoing open lung surgery: a randomized controlled trial. Korean J Anesthesiol. 2015;68:224–31. 26045924 10.4097/kjae.2015.68.3.224 PMC4452665

[14] Mohler JL Michael KA Freedman AM Griffen WO Jr McRoberts JW. The serum and urinary cortisol response to operative trauma. Surg Gynecol Obstet. 1985;161:445–9. 4049215

[15] Chernow B Alexander HR Smallridge RC. Hormonal responses to graded surgical stress. Arch Intern Med. 1987;147:1273–8. 3606284

[16] Udelsman R Norton JA Jelenich SE. Responses of the hypothalamic-pituitary-adrenal and renin-angiotensin axes and the sympathetic system during controlled surgical and anesthetic stress. J Clin Endocrinol Metab. 1987;64:986–94. 3031124 10.1210/jcem-64-5-986

[17] Donald RA Perry EG Wittert GA. The plasma ACTH, AVP, CRH and catecholamine responses to conventional and laparoscopic cholecystectomy. Clin Endocrinol (Oxf). 1993;38:609–15. 8392916 10.1111/j.1365-2265.1993.tb02142.x

[18] Koo BW Oh AY Ryu JH. Effects of deep neuromuscular blockade on the stress response during laparoscopic gastrectomy: randomized controlled trials. Sci Rep. 2019;9:12411. 31455832 10.1038/s41598-019-48919-2 PMC6711963

